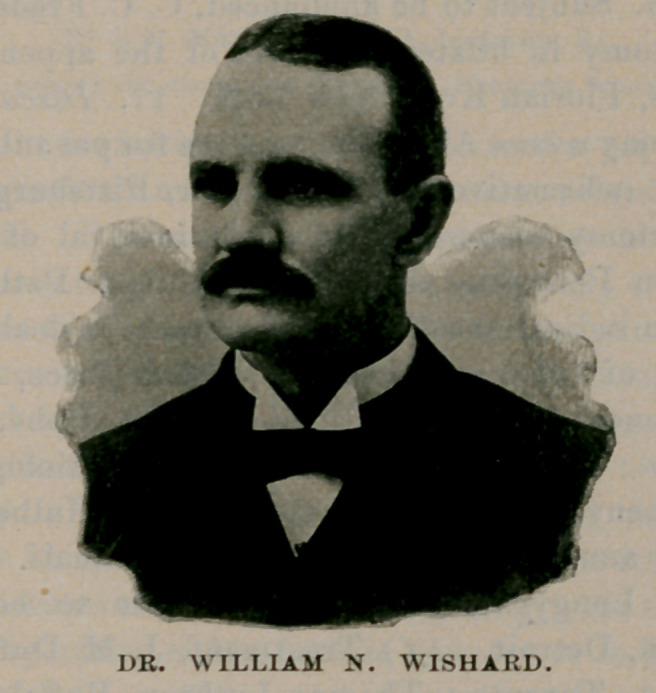# Society Meetings

**Published:** 1895-08

**Authors:** 


### Society Meetings.

The American Laryngological Association held its seventeenth annual meeting in Rochester, June 17, 18 and 19, 1895, under the presidency of Dr. John O. Roe. This was one of the best meetings in the history of this famous society. Dr. William H. Daly, of Pittsburg, was elected president for the ensuing year.

The American Orthopedic Association will hold its ninth annual meeting at Chicago, September 17, 18 and 19, 1895, under the presidency of Dr. John Ridlon, of Chicago. Dr. Bernard Bartow, of Buffalo, the first vice-president of the association, will read a paper on Forcible correction and corrective jackets in the treatment of scoliosis; and Dr. Roswell Park is announced for a paper on The deformities produced by acute inflammatory lesions in bone.

---

THE Mississippi Valley Medical Association will hold its twenty-first annual meeting at Detroit, September 3, 4, 5 and 6, 1895, under the presidency of Dr. William N. Wishard, of Indianapolis. A preliminary program containing the announcement of about sixty papers has been issued by the secretary, Dr. F. C. Woodburn, of Indianapolis.

DR. WILLIAM N. WISHARD.

The meeting promises to be a large one and ample arrangements for its scientific work and social festivities are preparing under the leadership of Drs. H. O. Walker, Eugene Smith and others. Detroit is within such easy reach of Buffalo, that a goodly number of physicians from this city can reasonably be expected to attend.

---

PRELIMINARY program of the American Association of Obstetricians and Gynecologists, eighth annual meeting at Auditorium Hotel, Chicago, September 24, 25 and 26, 1895: 1. President's annual address, J. Henry Carstens, Detroit. 2. Relation of pelvic suppuration to structural changes that may occur in the Fallopian tubes, A. P. Clarke, Cambridge. 3. Nephrorrhaphies, George Ben Johnston, Richmond. 4. Detached fibroids, George H. Rohé,

Catonsville. 5. A clinical contribution to lateral displacements of the uterus, Edward J. Ill, Newark. 6. Appendicitis, A. Vander Veer, Albany. 7. Intermediate treatment of puerperal sepsis, A. B. Miller, Syracuse. 8. Kraurosis Vulvæ, a contribution to its pathology and therapeutics, H. W. Longyear, Detroit. 9. Report of three recent cases in gall-bladder surgery, Edwin Ricketts, Cincinnati. 10. Subject to be announced, H. E. Hayd, Buffalo. 11. Intestinal obstruction following peritoneal operations, A. H. Cordier, Kansas City. 12. Subject to be announced, S. Y. Howell, Buffalo. 13. Cure of tubal distention without laparatomy, F. A. Glasgow, St. Louis. 14. Subject to be announced, W. B. Dorsett, St. Louis. 15. Subject to be announced, C. C. Frederick, Buffalo. 16. Hysterectomy in bilateral diseases of the appendages, giving remote results, Florian Krug, New York. 17. *Discussion:* Vaginal hysterectomy *versus* Abdominal section for pus tubes. (*a*) Title unannounced, (affirmative,) X. O. Werder, Pittsburg. (*b*) When shall hysterectomy accompany bilateral removal of the appendages? Reuben Peterson, Grand Rapids. (*c*) Pathological and surgical contra-indications of the vaginal route in dealing with puriform diseases of tubes and ovaries, Joseph Price, Philadelphia. (*d*) Title unannounced, (affirmative,) Geo. H. Rohé, Catonsville. 18. *Discussion:* Eclampsia gravidarum. (*a*) Etiology, Frederick Blume, Allegheny. (*b*) Pathology, George F. Hulbert, St. Louis. (*c*) Title to be announced, W. H. Taylor, Cincinnati. (*d*) Prophylaxis, H. W. Longyear, Detroit. (*e*) Title to be announced, W. P. Manton, Detroit. (*f*) Treatment, J. M. Duff, Pittsburg; A. H. Wright, Toronto; Thomas Lothrop, Buffalo. 19. Exhibition of various types of rectal papillæ, R. T. Morris, New York. 20. Subject to be announced, E. Arnold Praeger, Los Angeles, Cal. 21. Ruptured interstitial pregnancy, L. H. Dunning, Indianapolis. 22. Has gynecology received just recognition as a specialty? M. B. Ward, Topeka. 23. Subject to be announced, L. S. McMurtry, Louisville. 24. Pneumo-peritoneum, James F. W. Ross, Toronto. 25. Subject to be announced, J. B. Murphy, Chicago. 26. Subject to be announced, Charles A. L. Reed, Cincinnati. 27. Subject to be announced, M. Rosenwasser, Cleveland.

The regular program will be issued September 1st.

J. HENRY CARSTENS, *President.*

WILLIAM WARREN POTTER, *Secretary.*

## Figures and Tables

**Figure f1:**